# Gesture–speech physics in fluent speech and rhythmic upper limb movements

**DOI:** 10.1111/nyas.14532

**Published:** 2020-12-18

**Authors:** Wim Pouw, Lisette de Jonge‐Hoekstra, Steven J. Harrison, Alexandra Paxton, James A. Dixon

**Affiliations:** ^1^ Center for the Ecological Study of Perception and Action University of Connecticut Storrs Connecticut; ^2^ Donders Institute for Brain, Cognition and Behaviour Radboud University Nijmegen Nijmegen the Netherlands; ^3^ Institute for Psycholinguistics Max Planck Nijmegen Nijmegen the Netherlands; ^4^ Faculty of Behavioral and Social Sciences University of Groningen Groningen the Netherlands; ^5^ Royal Dutch Kentalis Sint‐Michielsgestel the Netherlands; ^6^ Department of Kinesiology University of Connecticut Storrs Connecticut; ^7^ Department of Psychological Sciences University of Connecticut Storrs Connecticut

**Keywords:** hand gesture, speech production, speech acoustics, biomechanics, entrainment

## Abstract

It is commonly understood that hand gesture and speech coordination in humans is culturally and cognitively acquired, rather than having a biological basis. Recently, however, the biomechanical physical coupling of arm movements to speech vocalization has been studied in steady‐state vocalization and monosyllabic utterances, where forces produced during gesturing are transferred onto the tensioned body, leading to changes in respiratory‐related activity and thereby affecting vocalization F0 and intensity. In the current experiment (*n* = 37), we extend this previous line of work to show that gesture–speech physics also impacts fluent speech. Compared with nonmovement, participants who are producing fluent self‐formulated speech while rhythmically moving their limbs demonstrate heightened F0 and amplitude envelope, and such effects are more pronounced for higher‐impulse arm versus lower‐impulse wrist movement. We replicate that acoustic peaks arise especially during moments of peak impulse (i.e., the beat) of the movement, namely around deceleration phases of the movement. Finally, higher deceleration rates of higher‐mass arm movements were related to higher peaks in acoustics. These results confirm a role for physical impulses of gesture affecting the speech system. We discuss the implications of gesture–speech physics for understanding of the emergence of communicative gesture, both ontogenetically and phylogenetically.

## Graphical abstract

The biomechanical physical coupling of arm movements to speech vocalization has been studied in steady‐state vocalization and monosyllabic utterances. We extend this previous work to show that gesture–speech physics also impacts fluent speech, and we discuss the implications of gesture–speech physics for understanding of the emergence of communicative gesture, both ontogenetically and phylogenetically.

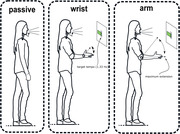

## Introduction

Communicative hand gestures are ubiquitous across human cultures. Gestures aid communication by seamlessly interweaving relevant pragmatic, iconic, and symbolic expressions of the hands together with speech.^1^, ^2^, ^3^ For such multiarticulatory utterances to do their communicative work, gesture and speech must be tightly temporally coordinated to form a sensible speech–gesture whole. In fact, the salient moments of gestures are often timed with emphatic stress made in speech, no matter what the hands depict.^4^, ^5^ For such gesture–speech coordination to get off the ground, the system must functionally constrain its degrees of freedom;^6^ in doing so, it will have to utilize (or otherwise account for) intrinsic dynamics arising from the biophysics of speaking and moving at the same time. Here, we provide evidence that movement of the upper limbs constrains fluent self‐generated speech acoustics through biomechanics.

### The gesture–speech prosody link

The tight coordination of prosodic aspects of speech with the kinematics of gesture has been long appreciated and is classically referred to as the beat‐like quality of cospeech gesture.^7^ As obtained from video analysis, gesture apices are often found to align with *pitch accents*—accents that are acoustically predominantly defined by positive excursions in the fundamental frequency (F0), lowering of the second formant, longer vowel duration, and increased intensity.^8^, ^9^, ^10^ Pitch accents can be perceptually differentiated by sudden lowering of F0 as well, but gestures do not seem to align with those events quite as much.^11^

More recent motion‐tracking studies have also found gesture–speech prosody correlations. For example, the peak velocity of gestures often co‐occurs near peaks in F0, even when such gestures are depicting something.^12^, ^13^, ^14^, ^15^, ^16^ In pointing gestures, stressed syllables align neatly with the maximum extension of the pointing movement, such that the hand movement terminates at the first syllable utterance in strong‐weak stressed “PA‐pa” and terminates later during the second syllable utterance in the weak‐strong “pa‐PA.”^17^, ^18^ During finger tapping and monosyllabic utterances, when participants are instructed to alternate prominence in their utterances (“pa, PA, pa, PA”), the tapping action spontaneously aligns with the syllable pattern, such that larger movements are made during stressed syllables.^19^ Conversely, if participants are instructed to alternate stress in finger tapping (strong, weak, strong, weak force production), speech will follow, with larger oral‐labial apertures for stressed versus unstressed tapping movements.

Even when people do not intend to change the stress patterning of an uttered sentence, gesturing concurrently affects speech acoustics in a way that makes it seem intentionally stressed, inducing an increase in vocalization duration and a lowering of the second formant of co‐occurrent speech.^20^ Furthermore, gesture and speech cycle rates seem to be attracted toward particular (polyrhythmic) stabilities: in‐phase speech‐tapping is preferred over antiphase coordination, and 2:1 speech‐to‐tapping ratios are preferred over more complex integer ratios such as 5:2.^21^, ^22^, ^23^, ^24^ This is similar to the research showing rhythmic stabilities arising out upper limb movement and their interactions with respiration cycles (e.g., Refs. 25 and 26). Thus, the upper limb and speech systems naturally couple their activity, like many other living as well as nonliving oscillatory systems^27^ (also see Ref. 28), requiring further study on the exact nature of this coupling.

### Gesture–speech physics

Mainstream understanding of the gesture–prosody link holds that it is not “biologically mandated” (p. 69 in Ref. 9; Ref. 29), requiring neurocognitive timing mechanisms^30^, ^31^ that appear only after about 16 months of age^32^ (see also Ref. 33). Recent work, however, has investigated a potential physical coupling of arm movements with speech via myofascial tissue biomechanics. This works shows that hand gesturing physically impacts steady‐state vocalizations and monosyllabic consonant‐vowel utterances.^34^, ^35^, ^36^, ^37^ Specifically, hand and arm movements can transfer a force (a physical impulse) onto the musculoskeletal system, thereby modulating respiration‐related muscle activity, leading to changes in the intensity of vocalization. If vocal‐fold adjustments do not accommodate the gesture‐induced impulses, the fundamental frequency (F0) of vocalizations is affected as well. Higher‐impulse arm movements or two‐handed movements will induce more pronounced effects on F0 and intensity than lower‐impulse wrist movements or one‐handed movements. This is because the mass of the “object” in motion is greater in magnitude for arm versus wrist movements, thereby changing the momentum of the effector (everything else—such as effector speed—being equal, as effector momentum equals effector mass times effector velocity). The change in momentum is the physical impulse, and physical impulse is highest when the change in velocity (i.e., acceleration) is highest (everything else—such as effector mass—being constant).

How physical impulses are absorbed by the respiratory system is likely complex and not a simple linear function.^38^ However, a complete understanding will involve an appreciation of the body as a prestressed system,^39^, ^40^ forming an interconnected tensioned network of compressive (e.g., bones) and tensile elements (e.g., fascia and muscles) through which forces may reverberate nonlinearly.^41^, ^42^ Specifically, the upper limb movements are controlled by stabilizing musculoskeletal actions of the scapula and shoulder joint, which directly implicate accessory expiratory muscles that also stabilize scapula and shoulder joint actions (e.g., the serratus anterior inferior; see Ref. 37 for an overview).

Peripheral actions also play a role, as performing an upper limb movement recruits a whole kinetic chain of muscle activity around the trunk (e.g., the rectus abdominis) to maintain posture.^43^, ^44^, ^45^ Indeed, when people are standing versus sitting, for example, the effects of peak physical impulse of gestures onto vocalization acoustics are more pronounced.^34^ We reasoned that this is because standing involves more forceful anticipatory postural counter adjustments,^46^ which reach the respiratory system via accessory expiratory muscles also implicated in maintaining postural integrity (see also Refs. 44 and 45). Recently, more direct evidence has been found for the gesture–respiration–speech link: respiratory‐related activity (measured with a respiratory belt) was enhanced during moments of peak impetus of gesture as opposed to other phases in the gesture movement, and respiratory‐related activity itself was predictive of the gesture‐related intensity modulations of monosyllablic utterances.^37^

The evidence reviewed so far has been based on experiments on continuous vocalizations or monosyllabic utterances and cannot, therefore, be directly generalized to fluent, self‐generated, full‐sentenced speech. However, recent work suggests that gesture–speech physics does generalize to fluent speech. For example, Cravotta and colleagues^47^ found that encouraging participants to gesture during cartoon narration versus giving no instructions led to a 22‐Hz increase in observation of max F0 and to greater F0 ranges of speech and intensity. Furthermore, computational modelers have reported interesting successes in synthesizing gesture kinematics on the basis of speech acoustics alone,^48^, ^49^ indicating that information about body movements inhabits the speech signal (see also Refs. 50 and 51). Although such results do not necessitate a role for biomechanics, they do suggest a strong connection between gesture and speech.

### Current experiment

The current experiment was conducted as a simple test of the constraints of upper limb movement on fluent speech acoustics. Participants were asked to retell a cartoon scene that they had just watched, while either not moving, vertically moving their wrist, or vertically moving their arm at a tempo of 80 beats per minute (1.33 Hz). Participants were asked to give a stress or beat in the downward motion with a sudden stop at maximum extension (i.e., sudden deceleration). Participants were asked to not allow movements to affect their speaking performance in any way. Similar to previous experiments,^34^, ^37^ we assessed the following to conclude that gesture–speech physics is present:

1. Does rhythmic cospeech movement change acoustic markers of prosody (i.e., F0 and amplitude envelope)?
2. At what moments of cospeech movement is change in acoustics observed?
3. Does degree of physical impulse (as measured by effector mass or changes in speed) predict acoustic variation?

## Materials and methods

### Participants and design

A total of 37 undergraduate students at the University of Connecticut were recruited as participants (mean age = 18.76, SD of age = 0.95, % cisgender female = 67.57, % cisgender male = 32.43, % right‐handed = 94.59).

The current design was fully within‐subject, with a three‐level movement manipulation (passive versus wrist‐movement versus arm‐movement condition). Movement condition was randomly assigned per trial. Taken together, participants performed 419 trials, each lasting about 40 seconds. The study design was approved by the IRB committee of the University of Connecticut (#H18‐227).

### Material and equipment

#### Cartoon vignettes

Twelve cartoon vignettes were created from the “Canary Row” and “Snow Business” Tweety and Sylvester cartoons (mean vignette duration = 59.42 s; SD = 32.11 s). These cartoons are often used in gesture research.^7^ The videos can be accessed here: https://osf.io/rfj5x/.

#### Audio and motion tracking

A MicroMic C520 cardioid condenser microphone headset (AKG, Inc.) was used to record audio at 44.1 kHz. The microphone was plugged into a computer that handled the recording via a C^++^ script. Also plugged into this computer was a Polhemus Liberty motion tracking system (Polhemus, Inc.), which tracked position of the participant's index finger of the dominant hand, sampling with one 6D sensor at 240 Hz. We applied a first‐order Butterworth filter at 30 Hz for the vertical position (z) traces and its derivatives.

### Procedure

**Figure 1:**
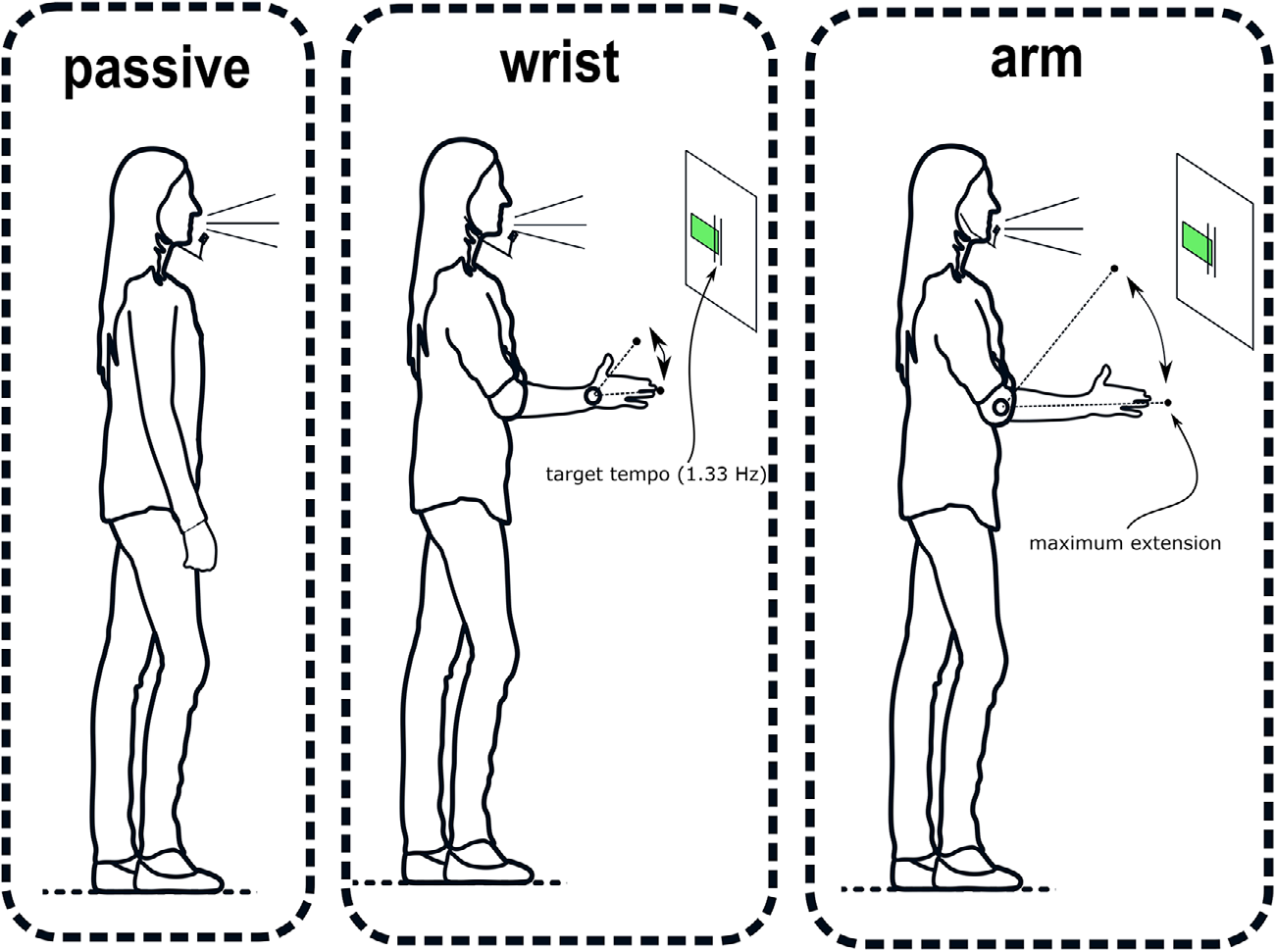
Graphical overview of movement conditions. Movement conditions are shown. Each participant performed all conditions (i.e., within‐subjects). To ensure that movement tempo remained relatively constant, participants were shown a moving green bar that indicated whether they moved too fast or too slow relative to a 20% target region of 1.33 Hz. Participants were instructed to have an emphasis in the downbeat with an abrupt stop (i.e., beat) at the maximum extension. The human pose figures were obtained and modified from an open database.^85^

Upon arrival, participants were briefed that this 30‐min experiment entailed retelling cartoon scenes while standing and performing upper limb movements. A motion sensor was attached to the tip of the index finger of their dominant hand, and a microphone headset was put on. Participants were asked to stand upright and were introduced to three movement conditions (Fig. 1). In the passive condition, participants did not move and kept their arm resting alongside the body. In the wrist‐movement condition, participants were asked to continuously move the hand vertically at the wrist joint while keeping the elbow joint at 90 degrees. In the arm‐movement condition, participants moved their arm vertically at the elbow joint, without wrist movement. Similar to previous studies,^34^ participants were asked to give emphasis in the downward motion of the movement with a sudden halt—in other words, a beat—at the maximum extension of their movement.

After introduction of the movements, participants were told that they were to move at a particular tempo, indicated by a visual feedback system. The feedback system consisted of a horizontal bar that continually updated to report on the participant's movement speed in the previous movement cycle. The participant was to keep the horizontal bar between the lower and higher boundaries (a 20% region, 72–88 BPM) of the 1.33‐Hz target tempo (i.e., 80 BPM). Participants briefly practiced moving at the target rate before starting the experiment. Critically, the participants were not exposed to an external rhythmic signal, like a visual metronome.

Subsequently, participants were instructed that they would watch and then retell cartoon clips while making one of the instructed movements (or making no movements). Participants were asked to keep their speech as normal as possible while making the movements (or no movement). In the conditions requiring movement, participants were to keep their movement tempo within the target range. Twelve cartoon vignettes were readied to be shown before each trial. The experiment ended when the participants saw and retold all 12 vignettes or when the total experiment time reached 30 minutes. To ensure that all movement conditions would be performed at least once within that time, we set the maximum time per trial at 1 minute. In other words, when participants were still retelling the same scene after 60 s, the experimenter would terminate the trial and move to the next trial. Mean retelling time was, however, well below 1 min (mean = 26.00 s, SD = 7.06 s).

### Preprocessing

#### Speech acoustics

The fundamental frequency was extracted with sex‐appropriate preset ranges (male = 50–400 Hz; female = 80–640 Hz). We used a previously written R script (https://osf.io/m43qy/)^52^ utilizing the R package “wrassp,”^53^ which applies a K. Schaefer–Vincent algorithm. It should be noted that F0 tracking is always susceptible to noisy estimation. We have, however, checked multiple participants’ data for mistrackings of the F0 algorithm (e.g., sudden jumps to higher harmonics) and did not find any. Given the current sample size, we did not hand‐check the F0 track for all the data, so we must accept a certain range of noise that is common to F0 tracking.

**Figure 2:**
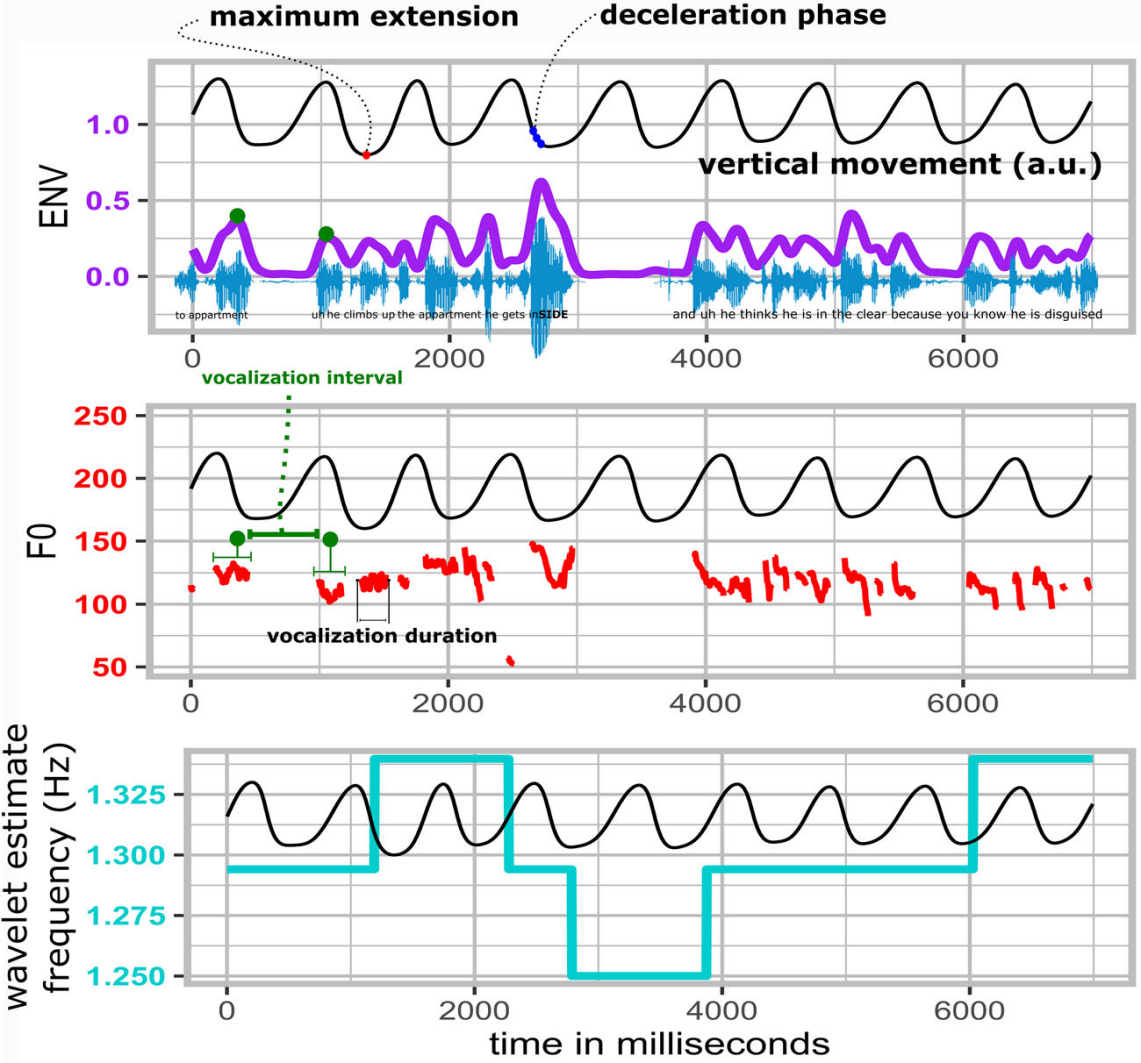
Example movement, amplitude envelope, F0 time series, and time‐dependent movement frequency estimates. A sample of about 10 s is shown. With the participant's permission, the speech sample is available at https://osf.io/2qbc6/. The smoothed amplitude envelope in purple traces the waveform maxima. The F0 traces show the concomitant vocalizations in Hz, with an example of vocalization interval and vocalization duration (which were calculated for all vocalizations). The bottom panel shows the continuously estimated movement frequency in cyan, which hovers around 1.33 Hz. In all these panels, the co‐occurring movement is plotted in arbitrary units (a.u.) to show the temporal relation of movement phases and the amplitude envelope, F0, and the movement frequency estimate. In our analysis, we refer to the maximum extension and deceleration phases as relevant moments for speech modulations. In this example, a particularly dramatic acoustic excursion occurs during a moment of deceleration of the arm movement, possibly an example of gesture–speech physics.

We also extracted a smoothed (5‐Hz Hann window) amplitude envelope using a previously custom‐written R script (https://osf.io/uvkj6/, which reimplements a procedure from Ref. 54). The amplitude envelope was calculated by applying a Hilbert transformation to the sound waveform, yielding a complex‐valued analytic signal from which we take the complex modulus. After smoothing and downsampling to 240 Hz, this gives a one‐dimensional time series referred to as the amplitude envelope, tracing the extrema of the sound waveform, as shown in Figure 2.

#### Data and exclusions

We collected 189.70 min of continuous data (passive condition = 63.45 min, wrist‐movement condition = 63.56 min, and arm‐movement condition = 62.69 min). However, a C^++^ memory allocation error caused insufficient storage to be reserved for more than 6 digits, which resulted in the loss of the precise timing information of the sampling of the motion tracker after a certain period, that is, after a seventh digit was needed to represent time (>1 million ms or 16 min and 40 s); fortunately, this affected only a subset of the experimental data for each participant. Full data were, therefore, obtained for the first 16 min and 40 s of each trial for each participant. We limited our analyses to this complete dataset. This dataset consists of 124.49 min of continuous speech and movement data (passive condition = 40.08 min, wrist‐movement condition = 42.32 min, and arm‐movement condition = 42.10 min).

### Baseline

We created a surrogate condition as a baseline for temporal coordination between speech and movement. We randomly paired the speech of the passive condition trials of participant *x* with motion‐tracking data from the movement conditions for that participant *x* (without scrambling the order of the speech and motion time series extracted in these falsely paired trials). This surrogate randomly paired condition allowed us to exclude the possibility that any effects of movement were due to chance correlations inherent to the structure of speech and movement, rather than the correlations arising out of the coupling of speech and movement. We only use this surrogate control condition as a contrast when we are performing analysis on the temporal relation between speech and movement.

### Manipulation checks

We computed additional measures to check whether our movement manipulation was successful and whether speech rates were comparable across conditions. Figure 2 shows a summary of the results for key manipulation check measures.

#### Movement frequency

To ascertain whether participants moved their limbs within the target 1.33‐Hz range, we performed a wavelet‐based analysis (using the R package “WaveletComp”^55^). Wrist movements were performed at slightly faster rates (mean = 1.44 Hz, SD = 0.24) than arm movements (mean = 1.36 Hz, SD = 0.19), but in both cases, the movements were distributed over the target range. This confirms that our movement manipulation was successful. For our surrogate control condition, the mean frequency of the artificially paired movement time series fell between both arm‐ and wrist‐movement condition frequency distributions (mean = 1.41 Hz, SD = 0.22).

#### Speech rate

We calculated two measures of speech rate: vocalization duration and vocalization interval (see Fig. 2 for examples), which are measures derived from information in the F0 track, as well as the amplitude envelope for the interval calculation. The vocalization duration was defined as the length of time (in milliseconds) of an uninterrupted run of F0 observations. The vocalization interval was determined by identifying two consecutive runs of F0 observations (i.e., vocalization events) and determining the peak amplitude envelope of each of those vocalization events so as to compare the relative timing between those peaks. This way we have a single time point for each vocalization event that we can compare with the next vocalization event's time point (i.e., the vocalization interval).

**Figure 3:**
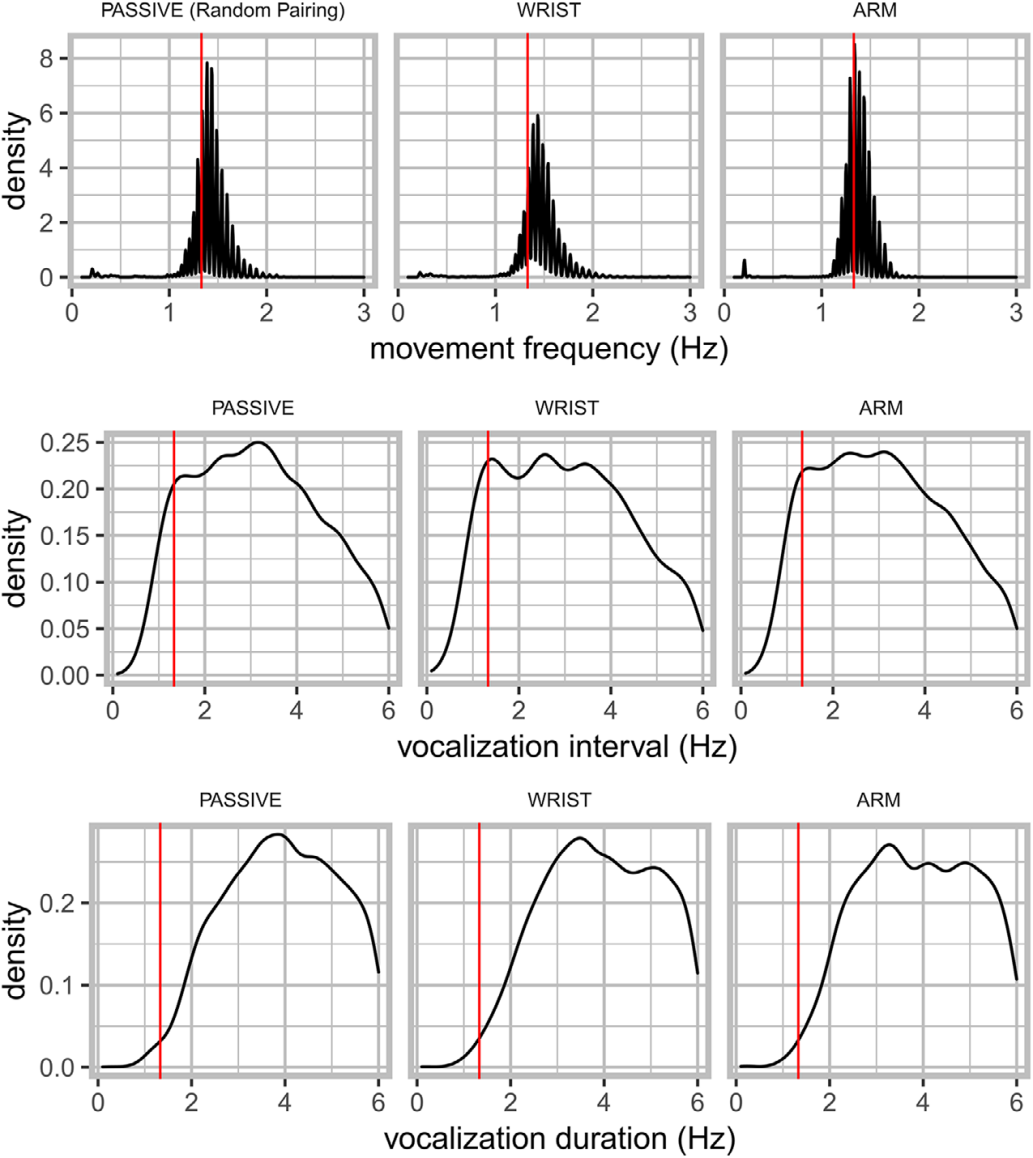
Summaries of movement frequency, vocalization duration, and vocalization interval. Density distributions of movement frequencies, vocalization interval, and vocalization duration are shown. There was no movement for the passive condition, but we display the randomly paired movement time series in the surrogate baseline pairing for which frequency information is shown. The red vertical line indicates the target movement frequency (1.3 Hz).

Figure 3 shows relatively uniform distributions for these specific speech measures. No clear 1:1 frequency couplings of movement and vocalization duration or vocalization interval nor any other clear signs of polyrhythmic coupling of movement and speech are observed (see, e.g., Refs. 22 and 24). Note, though, that there are other possible (acoustically defined) units of speech that might entrain to movements that we do not further pursue here.^56^ We restrict ourselves for the current report to speech vocalization acoustics rather than speech–movement cycle dynamics, as the former is the confirmatory research topic of the current study.

To compare vocalization rates with movement, we computed the average vocalization duration and interval for each trial by tracking the time of uninterrupted runs of F0 observations and then converting the time in milliseconds to hertz. For the passive condition, the average vocalization duration was 6.28 Hz (SD = 6.03), and the average vocalization interval was 5.17 Hz (SD = 6.94). For the wrist‐movement condition, the average vocalization duration was 6.24 Hz (SD = 5.96), and the average vocalization interval was 5.02 Hz (SD = 6.86). For the arm‐movement condition, the average vocalization duration was 6.08 Hz (SD = 5.83), and the average vocalization interval was 4.86 Hz (SD = 5.76).

### Availability of data and analyses

All anonymized data and analysis code are available at the Open Science Framework (https://osf.io/tgbmw/). This manuscript has been written with Rmarkdown; for the code‐embedded reproducible version of this manuscript, please see the Rmarkdown (.Rmd) file available at the OSF page.

## Results

### Overview of analyses

We report three main analyses to show that gesture–speech physics is present in fluent speech. First, we assess the overall effects of movement condition on vocalization acoustics (F0 and the amplitude envelope); these would support our hypothesis that upper limb movement—especially high‐impulse movement—constrains fluent speech acoustics. Second, we assess whether vocalization acoustic modulations are observed at particular phases of the movement cycle, which gesture–speech physics holds should occur at moments of peaks in deceleration. Third, we assess whether a continuous estimate of upper limb physical impulse through deceleration rate predicts vocalization acoustic peaks, which would support the gesture–speech physics hypothesis that physical impulses are transferred onto the vocalization system.

The following generally applies to all analyses. For hypothesis testing, we performed mixed linear regression models (using the R package “nlme”^57^), and nonlinear generalized additive modeling or GAM (using the R package “gam”^58^) with random intercept for participants by default.

### Acoustic correlates of the movement condition

**Figure 4:**
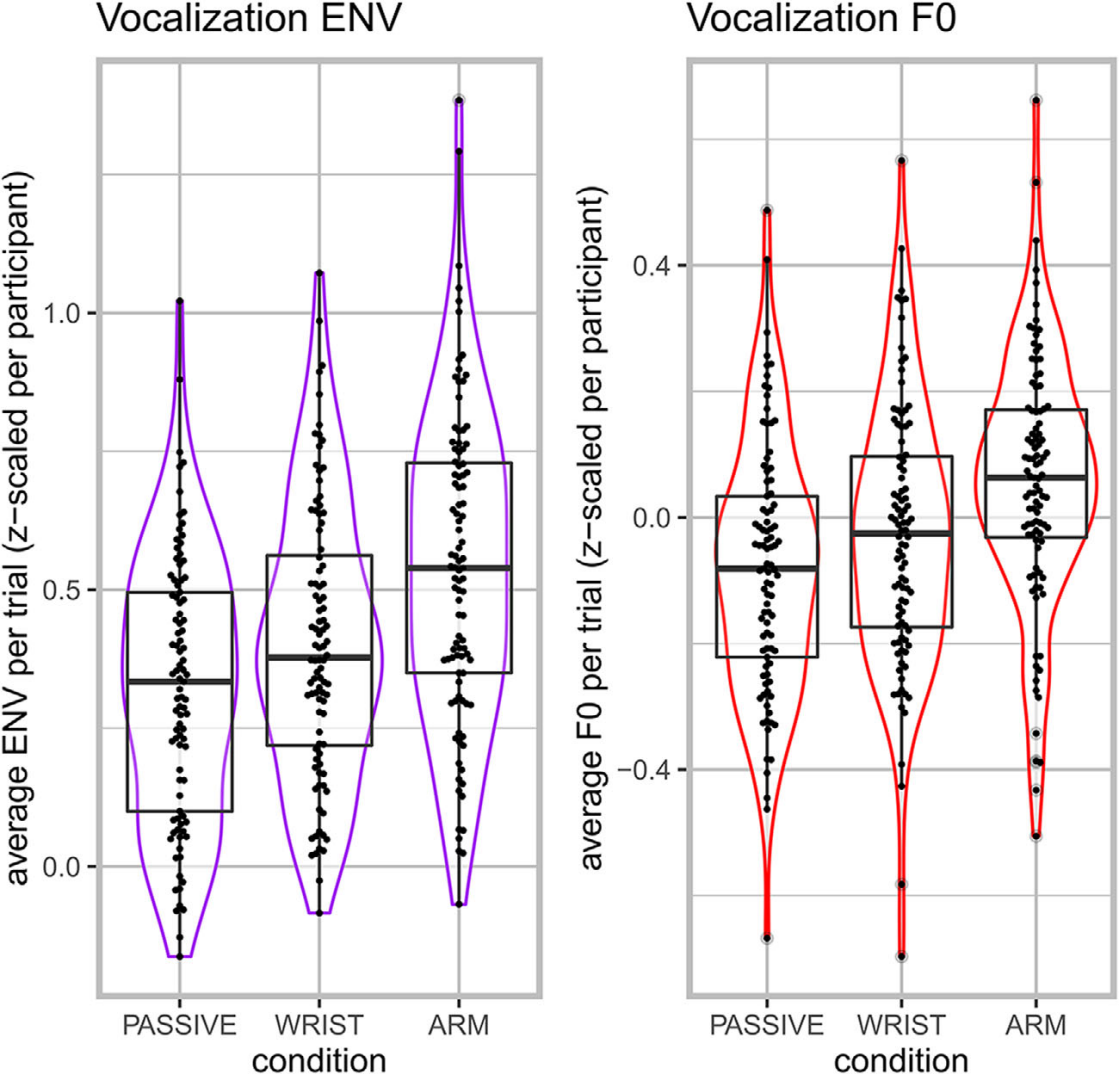
Average F0 and amplitude envelope (ENV) per trial per condition. Violin and box plots are shown for average F0 (Hz) and amplitude envelope (*z*‐scaled) per trial. (Points are jittered to show per‐trial observations).

Figure 4 shows the average F0 and amplitude envelope (*z*‐scaled for participants) per trial per condition. The passive condition had generally lower levels of F0 and amplitude envelope as compared with the arm‐ and wrist‐movement conditions. Furthermore, the higher‐impulse arm‐movement condition generally had higher levels of F0 and amplitude envelope as compared with the lower‐impulse wrist‐movement condition.

Table 1 shows the results of mixed linear regression analysis. For the amplitude envelope, the passive condition had a lower average amplitude envelope as compared with the wrist‐movement condition, as well as the arm‐movement condition. After accounting for differences in F0 for sex (males had generally 73 Hz lower F0), the wrist‐movement condition had about a 1.6‐Hz increase in average as compared with the passive condition, but this was not statistically significant. Furthermore, the arm‐movement condition increased F0 by 3.5 Hz over the passive condition.

**Table 1:** Linear mixed effects for effects of condition on F0 and amplitude envelope (ENV)

	Contrast	*b*	SE	*df*	*P*
ENV (*z*‐scaled)	Intercept	0.32	0.036	251	<0.0001
	Wrist versus passive	0.094	0.028	251	0.001
	Arm versus passive	0.215	0.028	251	<0.0001
F0 (Hz)	Intercept	186.577	3.22	251	<0.0001
	Male versus female	−73.268	5.437	33	<0.0001
	Wrist versus passive	1.603	0.845	251	0.0588
	Arm versus passive	3.504	0.828	251	<0.0001

### Coupling of vocalization duration and movement

**Figure 5:**
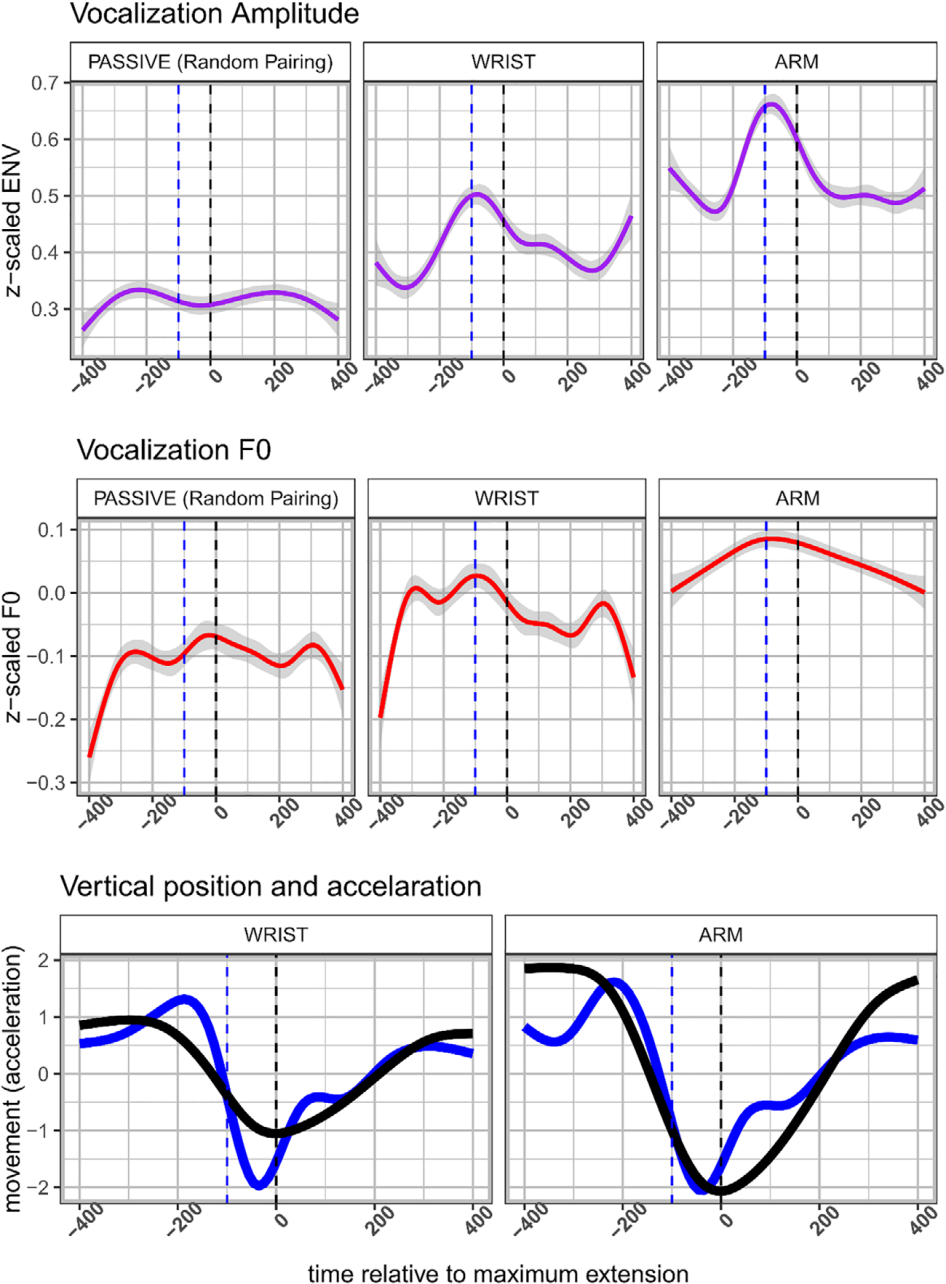
Average observed vocalization acoustics relative to the moment of maximum extension. For the upper two panels, the average acoustic trajectory is shown around the moment of maximum extension (*t* = 0, black vertical dashed line). In the lower panel, we have plotted the *z*‐scaled average vertical displacement of the hand and the *z*‐scaled acceleration trace. The blue vertical dashed line marks the moment where the deceleration phase starts, which aligns with peaks in acoustics.

Having ascertained in the previous analysis that acoustics were modulated for movement versus no movement, we further need to confirm that such modulations occur at particular moments in the movement cycle. Figure 5 shows the main results for all data, for which we model over time the acoustic patterning in vocalizations around the maximum extension of the movement cycle, for all movement cycles that occurred. If vocalizations are affected in particular moments of the movement cycle—for example, when the hand starts decelerating (estimated from the data as shown in Fig. 5)—we would expect acoustic modulations (peaks) at such moments of the movement cycle.

Just before the moment of maximum extension, the observed amplitude envelope shows a clear peak, most dramatically for the arm‐movement condition, but also for the wrist‐movement condition. For speech in the randomly paired movement and passive conditions, this was not the case; this provides evidence that the results observed in the arm‐ and wrist‐movement conditions are not due to mere chance. For F0, the pattern is somewhat less clear, but positive peaks still occur just before the maximum extension. These findings replicate our earlier work on steady‐state vocalization and monosyllabic utterances, showing that moments of peak deceleration show peaks in acoustics.^34^, ^37^

To test whether trajectories are indeed nonlinear and are reliably different from the passive condition, we performed GAM, a type of nonlinear mixed effects procedure. GAM is a popular time‐series analysis in phonetics and allows the automatic modeling of more (and less) complex nonlinear patterns by combining a set of smooth basis functions. Furthermore, GAM allows for testing whether those nonlinear trajectories are modulated depending on some grouping of the data (see, e.g., Ref. 59). We assessed the trajectory of acoustics around 800 ms of the maximum extension of the movement. We chose 800 ms (–400, 400), as this is about the duration of a 1.33‐Hz cycle (1000/1.33 Hz = 752 ms) with an added margin of error of about 50 milliseconds. The model results with random slopes and intercept for participants are shown in Table 2.

**Table 2:** Model results for GAM analysis

	Contrast	*b*	SE	*df*	*P*
ENV (*z*‐scaled)	Intercept	0.237	0.006	36.923	<0.0001
	Wrist versus passive	0.096	0.009	10.579	<0.0001
	Arm versus passive	0.152	0.009	16.862	<0.0001
F0	Intercept	−0.061	0.006	−8.35	<0.0001
	Male versus female	−0.019	0.009	−4.29	<0.0001
	Wrist versus passive	0.101	0.009	10.222	<0.0001
	Arm versus passive	0.094	0.103	9.546	<0.0001

Note: Model results are shown for the amplitude envelope (ENV; *z*‐scaled) and F0 (Hz). For F0, we accounted for sex differences when estimating independent effects of condition.

**Figure 6:**
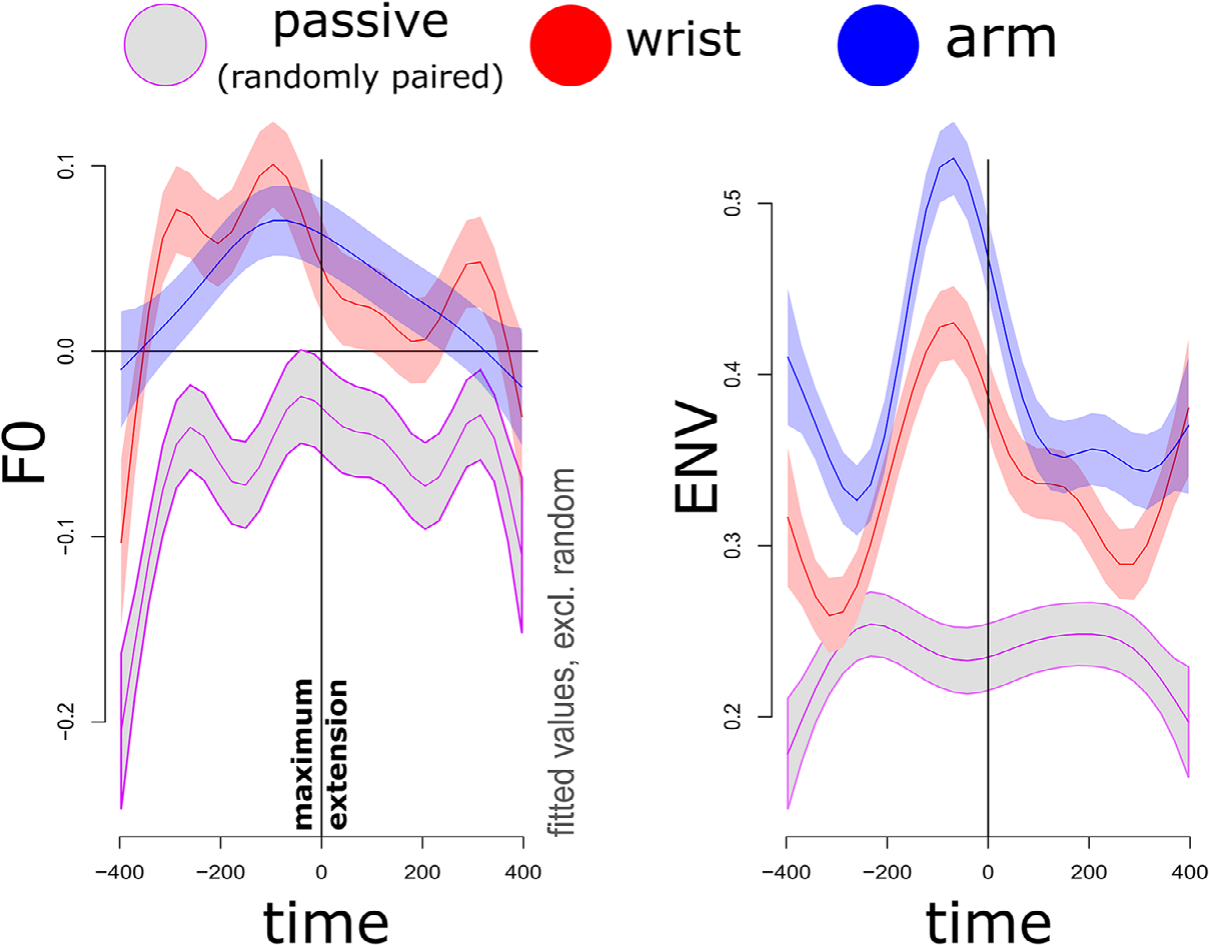
Fitted trajectories GAM.

First, for all models, tests for nonlinearity of the trajectories were statistically significant (*P*s < 0.0001), meaning that there were peaks or valleys in acoustics over the movement cycle rather than a flat linear trend (Fig. 6). As shown in Table 2, our results replicate the general finding that the wrist‐movement condition led to reliably different nonlinear peaks in acoustics as compared with the passive condition (*P* < 0.001). Moreover, this effect—relative to the passive condition—is even more extreme for the arm‐movement condition (*P* < 0.001). Figure 6 provides the fitted trajectories for the GAM models.

For readers interested in the individual differences in trajectories, we have created interactive graphs for each participant's average amplitude envelope trajectories (https://osf.io/a423h/) and F0 trajectories (https://osf.io/fdzwj/).

### Degree of physical impetus and acoustic peaks

We have confirmed that speech acoustics are modulated around moments of the deceleration phase, about 0–200 ms before the maximum extension. The effect of gesture–speech physics can be further examined by looking at how the forces produced by the upper limb movement predict acoustic peaks. Therefore, for all vocalizations that occurred between 200 and 0 ms before the maximum extension, we assessed whether the acoustic peak (i.e., maximum F0 or maximum amplitude envelope) was predicted by the maximum deceleration value (i.e., minimum acceleration observation) observed in that 200‐ms window. In a previous research, we found that higher deceleration was related to higher amplitude envelope observations but not F0.^37^

**Figure 7:**
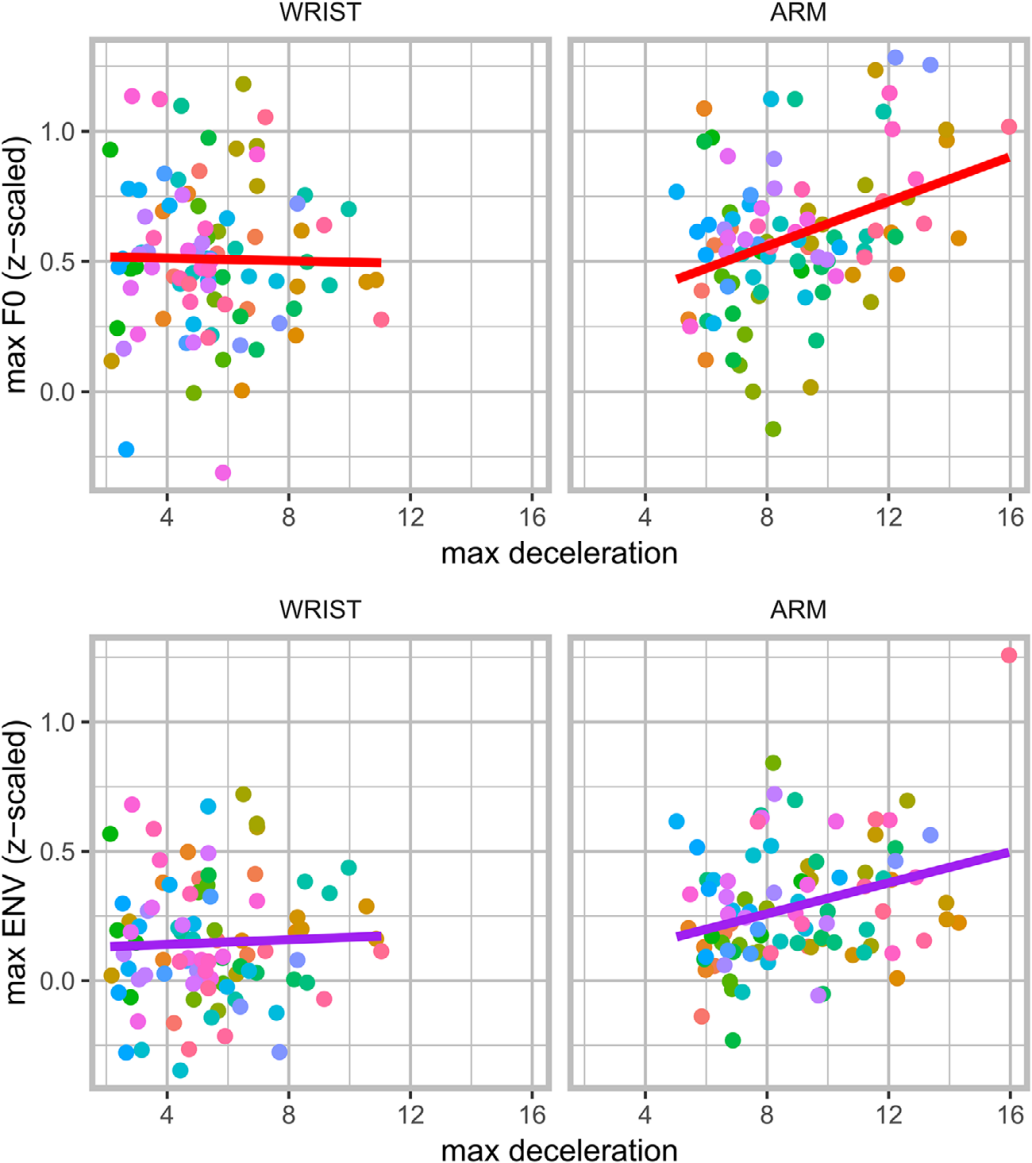
Relation between maximum deceleration and acoustic peak height. The *x*‐axis shows the average maximum deceleration per trial (absolutized negative acceleration value), where 0 indicates no deceleration (absolutized) and positive values indicate higher deceleration rates in cm/s^2^. Each point contains trial‐averaged values. It can be seen that deceleration rates are more extreme for the arm versus the wrist condition. On the *y*‐axis, we have the average maximum observed amplitude envelope (lower panel) and F0 (upper panel) for those moments of deceleration. Higher decelerations co‐occur with higher peaks in acoustics for arm movements (but not or less so for wrist movements).

Figure 7 shows the general pattern of the results for the wrist‐ and arm‐movement conditions. For each participant's trial in each condition, we averaged the maximum deceleration values of max F0 and max ENV for each vocalization event. Table 3 shows the results of a linear mixed‐effects model with random intercept and slopes for participants, in which we regressed the trial‐averaged maximum observed deceleration against the co‐occurring trial‐averaged vocalization acoustic peaks for amplitude envelope and F0 (separately). Higher deceleration indeed predicted higher amplitude envelope. This was also the case for F0, but only for arm movement (as opposed to wrist movement), as indicated by a statistically significant interaction between condition and max deceleration effect (*P* < 0.05). Together, these demonstrate the roles of both acceleration and effector mass in producing physical impulses.

**Table 3:** Linear mixed effects of deceleration and acoustic peaks

Model	Contrast	*b*	SE	*df*	*P*
1. ENV (*z*‐scaled)	Intercept	0.003	0.06	153	0.9597
	Max deceleration	0.029	0.007	153	<0.001
2. F0 (*z*‐scaled)	Intercept	0.512	0.086	151	<0.0001
	Arm versus wrist	−0.284	0.134	151	0.0349
	Max deceleration	−0.001	0.015	151	0.9603
	Arm × Max deceleration	0.042	0.018	151	0.0205

Note: Wrist movement is the reference factor for model 2.

## Discussion

In the current study, we demonstrated biomechanical effects of flexion‐extension upper limb movements on speech, thereby replicating in fluent speech effects obtained in steady‐state vocalization and monosyllabic utterances.^34^, ^35^, ^37^ We showed that rhythmically moving the wrist or arm affects vocalization acoustics by heightening the F0 and amplitude envelope of speech vocalizations, as compared with both passive‐ and statistical‐control conditions. We finally show that higher deceleration rates observed within 200 ms before the moment of the maximum extension of the arm movement materializes into more extreme acoustic peaks, demonstrating a role for acceleration and effector mass for the effect of gesture on speech (i.e., an effect of physical impulse). Indeed, in all analyses, we observe that higher‐mass arm versus wrist movements affect speech more clearly.

Thus, stabilities in speaking may arise out of gesture–speech biomechanics in fluent speech as well as more simplified speech sounds. This does not mean that speech prosody necessarily requires gesture for reaching prosodic targets. Indeed, other sensorimotor solutions are available for modulating F0 and intensity (e.g., vocal‐fold tensioning and respiratory actions^60^). Furthermore, F0 is uniformly less (if at all) affected, in line with our previous work^37^ and other work on the variable and often negligible role of respiratory actions in F0 modulations.^61^ However, we think that, on the basis of present work, we can argue that the biomechanical coupling of gesture and speech provides a “smart” mechanism for “timing” acoustic and movement expressions—and provides a way toward understanding the phylogenetic origin of the pulse or beat quality of gesture.

We should wonder still whether the current effects of upper limb movement can be produced due to attentional guidance to the movement (in the sense of “I must stop my wrist here and move up”), rather than the physical impulses produced by moving. In the previous studies, we provided additional evidence with a respiration belt that tensioning around the trunk is involved in gesture‐induced effects on vocal acoustics^37^ or that postural stability moderates said effects.^34^ The additional evidential strength of these previous studies for gesture–speech physics lies in part in that a cognitive control account does not (1) readily predict that trunk tensioning is involved in synchronizing upper limb movement and speech and (2) equally does not predict that standing or sitting matters for synchronizing speech and gesture trajectories. It should be noted here that trunk tensioning and postural control effects could be explained (in principle) with some new cognitive control account, but such an account would not seem parsimonious compared with a gesture–speech physics alternative.

This reasoning from parsimony also extends to the basic kinematic‐acoustic analysis of the current study. We should, therefore, ask in the current context: Does a cognitive control account predict that arm motion versus wrist motion should lead to heightened acoustic effects, that acoustic peaks arise around the deceleration phase rather at the maximum extension phase, or that the degree to which a limb in motion decelerates scales with the acoustic peak that ensues? It is wholly possible that a particular cognitive control theory may still account for all these effects or, more likely, a subset of these effects. But to do so, one needs to invoke some new hypothesis about how this cognitive control system produced these observables. This comes at the cost of parsimony, as we are invoking new unobservable mechanisms to explain these observables—especially if a more parsimonious theory that explains these effects is already available.

To be clear, this does not mean that we can fully exclude cognitive control—neither in principle nor, more forcefully, in degree. Fluid speech likely includes bidirectional interactions either of amplification or counteraction of gesture–speech physics with lexical, syntactic, and prosodic speech organization. In other words, complex interactions likely arise between the biophysical constraints arising out of moving your upper limb while vocalizing and a speech system organizing meaningful speech in the context of those constraints (see, e.g., Refs. 32 and 62). For example, a speaker might speed up the occurrence of a physical impulse, as then it will occur during a part of speech where there is a lexical stress. Or a speaker might counteract an F0 effect of a physical impulse laryngeally, as its acoustic effect would lead to an inappropriate acoustic marker in the syntactic context of the sentence. These potential interactions between gesture–speech physics and meaningful speech organization must be studied in controlled experiments, but we believe they likely also exist in real‐world contexts.

While future research should include controlled experiments on syntactic, lexical, and prosodic interactions with biophysical constraints, more research is needed on truly spontaneous speech as well. In the current study, participants are retelling a cartoon, which is a very different context than, say, a conversation; in part because the cognitive load of having to retell something accurately from recent memory while also having to move (see, e.g., Refs. 26, 63, and 64).

### Wider implications

Gesture–speech physics holds promise for revising our understanding of the emergence of communicative gesture in anatomically modern humans, both ontogenetically and phylogenetically.

It is well known that infants produce concurrent vocal–motor babblings. Furthermore, increased rhythmicity or frequency of motor babbling predicts speech‐like maturation of vocalization.^65^, ^66^ Rather than a primarily neural development that instantiates gesture–speech synchrony,^32^ we suggest that during such vocal–motor babblings, gesture–speech physics is discovered; this could provide the basis for infants to develop novel stable sensorimotor solutions for communication, such as a synchronized pointing gesture with a vocalization. Such sensorimotor solutions are, of course, likely solicited and practiced through the support of caretakers, yet without the biomorphological scaffolding, gesture–speech synchrony would not get off the ground ontogenetically.

Phylogenetic accounts have been central in discussions of the drivers of the depiction and referential function of gesture.^67^, ^68^, ^69^ However, the current work supports the view that peripheral body movements may have served as a control parameter of an evolving vocal system. Previous work has proposed that the vocal system may have been evolutionarily exapted from rhythmic abilities in the locomotor domain,^70^, ^71^ and viewing upper limb movements as constraints on the vocal system's evolution fits neatly in such views. When our species became bipedal, the respiratory system was thereby liberated from upper limb locomotor perturbations. We know that breathing (and vocalization) cycles often rigidly couple 1:1 with locomotion cycles in quadrupeds,^72^ rigidly limiting what can be done (or communicated) in one breath. Similarly, the vocalization acoustics of flying bats are synchronized with their wing beats through respiratory interactions.^73^ Bipedalism, however, did not only free respiration from locomotion; it freed the upper limbs, too, allowing these highly skilled articulators to modulate a possibly less skilled respiratory‐vocal system. Gestures, then, may have played a role in the complexification of the control of the respiratory system in our species, which has been attributed to have occurred to serve speech evolution.^74^, ^75^

Upper limb–vocal synchrony is not specific to human culture, as many nonhuman animals can also do it (e.g., bats^73^). It can further be related to other species, including orangutans, who deepen their vocalizations by cupping their hands in front of their mouths.^76^ Other animals have been found to be sensitive to body‐related information in sound in that body size and strength can be detected from vocalizations alone,^77^, ^78^ and humans are able to do this with some accuracy as well,^79^ even when they are blind from birth.^80^ In a recent experiment, we found that listeners are exquisitely sensitive to gesture‐modulated acoustics: listeners can synchronize their own upper limb movements by simply listening to vocalizers producing steady‐state vocalizations while rhythmically moving their wrists or arms.^35^ Thus, bodily dynamics can imprint the (human) voice, and this can be informative for listeners. Further research is needed to see if possibly other bodily contexts can tune and live through the vocal system similarly as hand gestures, for example, head gesturing and body postures.^81^, ^82^, ^83^

To conclude, gesture–speech physics opens up the possibility that gesture may have evolved as a control parameter on vocal actions. This ecological revision^42^, ^84^ of gesture–speech coupling provides a solid phylogenetic basis for a coevolution of gesture and speech, whereby peripheral bodily tensioning naturally formed coalitions with sound‐producing organs that were still very much under development.

## Author contributions

W.P. designed the study, conducted the research, analyzed the data, together with crucial guidance of L.d.J.‐H., S.J.H., A.P., and J.A.D. at all stages of the research. W.P. wrote the manuscript, with critical revision by L.d.J.‐H., S.J.H., A.P., and J.A.D.

## Competing interests

The authors declare no competing interests.
